# Arthroscopy and arthrotomy were equally effective for medial meniscal release but arthroscopy was minimally advantageous at preventing iatrogenic damage to the caudal cruciate ligament: a canine cadaveric study

**DOI:** 10.3389/fvets.2025.1452969

**Published:** 2025-02-13

**Authors:** Dana N. Gale, Steven W. Frederick, Bianca F. Hettlich, Jennifer J. Au, Tatiana Motta

**Affiliations:** ^1^BluePearl Pet Hospital, Christiana, DE, United States; ^2^BluePearl Science, Marietta, GA, United States; ^3^Independent Consultant, Kleve, Germany; ^4^Surgery and Rehabilitation, New River Veterinary Specialists, Hardeeville, SC, United States; ^5^Veterinary Clinical Sciences, The Ohio State University College of Veterinary Medicine, Columbus, OH, United States

**Keywords:** dog, meniscus, stifle, arthroscopy, arthrotomy, cartilage, iatrogenic

## Abstract

**Objective:**

To compare efficacy of four techniques used for medial meniscal release by medial caudal meniscotibial ligament transection and evaluate associated iatrogenic damage to the medial meniscus, caudal cruciate ligament (CdCL), and articular cartilage of the canine stifle joint.

**Study design:**

Twenty-four pairs of canine cadaveric pelvic limbs were randomly assigned to groups by methods of approach, cranial tibial translation, and meniscal release. I: arthrotomy, Hohmann, #11 scalpel blade; II: arthrotomy, Hohmann, #64 Beaver blade; III: arthroscopy, Hohmann, meniscal hook knife; IV: arthroscopy, no joint translation, meniscal hook knife. Post-procedure stifle dissection and evaluation of meniscal release success rate and presence of iatrogenic damage were performed. Fisher’s exact tests were performed for meniscal release and damage comparisons. Iatrogenic damage to the CdCL and articular cartilage were compared using generalized linear mixed effects model and linear mixed effects models (G/LMM) respectively.

**Results:**

Incomplete meniscal release was identified in 0/12 (0%) stifles in group I, 1/12 (8.3%) stifles in group II, 2/12 (16.7%) stifles in group III, and 1/12 (8.3%) stifles in group IV (*p* = 0.89, Fisher’s exact test). There was no difference in iatrogenic meniscal damage rates between groups (*p* = 0.48, Fisher’s exact test). There were no differences in total surface area of iatrogenic articular cartilage damage in any tested region between groups: femoral trochlea (*p* = 0.32, LMM), femoral condyles (*p* = 0.54, LMM), tibia (*p* = 0.28, LMM). Group I had more iatrogenic damage to the CdCL than group IV (*p* = 0.04, GLMM); no other differences were found.

**Conclusion:**

Arthroscopy and arthrotomy were equally effective for performing medial meniscal release by transection of the medial caudal meniscotibial ligament. Arthroscopic evaluation and medial meniscal release without joint translation was minimally advantageous in preventing iatrogenic damage to the CdCL.

## Introduction

Medial meniscal injury can occur concurrently or secondarily in up to 77% of dogs with cranial cruciate ligament disease (CCLD) (1–7). Additionally, medial meniscal injuries have been reported as a secondary injury due to residual instability after stifle stabilization in 2.8–13.8% of CCLD dogs (5, 7–9), or as a failure of diagnosis at the time of stabilization surgery (2, 10).

Stifle arthroscopy and parapatellar arthrotomy are two commonly accepted approaches for direct visualization of the meniscus and initial steps in treatment of a damaged meniscus. Arthroscopy with probing of the medial meniscus is a minimally invasive procedure that offers enhanced visualization, increased accuracy in assessment of the medial meniscus and lower postoperative morbidity rates (11–14), and it is considered the gold standard for diagnosis of medial meniscal pathology (12–14). However, parapatellar arthrotomy requires less setup time and does not require expensive equipment or specialized training (12, 13).

Although arthroscopy and arthrotomy are widely used for inspection of the intraarticular structures of the stifle joint, iatrogenic articular cartilage injury has been reported to occur with both methods (15–17). Additionally, Austin et al. reported iatrogenic cartilage damage associated with meniscal release procedures in about 10% of cases (18), which was reportedly decreased with the use of a stifle distractor in small breed dogs (19).

The aims of this study were to objectively compare the efficacy of four techniques used for medial meniscal release by medial caudal meniscotibial ligament transection, to objectively measure iatrogenic articular cartilage damage associated with each method, and to subjectively evaluate iatrogenic ligament and meniscal damage with each method. The authors hypothesized that arthroscopic techniques for medial meniscal release would result in greater efficacy of meniscotibial ligament transection compared to non-arthroscopic techniques. The authors also hypothesized that arthroscopic techniques for meniscal release would result in less iatrogenic articular cartilage damage, as well as ligament and meniscal damage, compared to non-arthroscopic meniscal release techniques.

## Methods

### Study subjects

Canine cadaveric pelvic limbs (*n* = 25 pairs) were harvested from local shelter dogs euthanized for reasons unrelated to this study in accordance with American Veterinary Medical Association Guidelines for the Humane Euthanasia of Animals and approved by the Ohio State University College of Veterinary Medicine’s IACUC (Preclinical Models of General Surgery, Protocol number: 2013A00000125). Dogs were of various breeds, weighed between 19 to 37.5 kg, skeletally mature (ages unknown), and had no overt evidence of orthopedic disease based on palpation. The specimens were clipped to remove all hair from mid-thigh to mid-crus. Left and right pelvic limbs were harvested by coxofemoral disarticulation and circumferential soft tissues were preserved. Each limb was wrapped in a saline soaked towel and stored at −20°C for later use, at which time the limbs were slowly thawed at room temperature.

Each limb was randomly assigned to one of four experiment groups using a random number generator. Groups were as follows: group I (arthrotomy, crania tibial translation with a small Hohmann retractor, and meniscal release with an #11 scalpel blade), group II (arthrotomy, cranial tibial translation with a small Hohmann and meniscal release with a #64 Beaver blade), group III (arthroscopy, cranial tibial translation with a small Hohmann and meniscal release with a meniscal hook knife), or group IV (arthroscopy, no instrumented translation and meniscal release with a meniscal hook knife). An equal number of right and left limbs were assigned to each group and no pairs of limbs were assigned to the same group. All procedures were performed by two board certified surgeons (JA, BH) with clinical experience in stifle arthroscopy. For all procedures, pelvic limbs were positioned using a vice grip at the end of the operating table. The stifles were positioned at approximately 120° flexion.

### Medial parapatellar arthrotomy

A cranial medial parapatellar arthrotomy was performed as previously prescribed (20). A medial parapatellar curvilinear incision was made through the skin and subcutis from the caudomedial aspect of the tibial tuberosity and extending proximally to the base of the patella using a #10 scalpel blade. The medial fascia dissection was continued from the apex of the patella to the tibial tuberosity several millimeters medial to the patellar tendon. A stab incision was made into the joint capsule using an #11 scalpel blade. The incision was extended proximally using Mayo scissors, through the parapatellar fibrocartilages, medial fascia, and insertion of the vastus medialis muscle and cranial part of the sartorius muscle. The patella was luxated laterally. A Gelpi retractor of appropriate size was inserted into the arthrotomy to aid in visualization of the joint. The infrapatellar fat pad was retracted or excised if necessary to improve visualization. The cranial cruciate ligament (CrCL) was transected with an #11 scalpel blade. A small Hohmann retractor (220 mm by 8 mm, DePuy Synthes, West Chester, Pennsylvania) was inserted into the intercondylar notch to provide cranial tibial translation. The medial meniscus was inspected using a meniscal probe (Small Joint Hook Tip Probe, Arthrex® Vet Systems, Naples, Florida). For specimens in group I, medial meniscal release was performed via transection of the medial caudal meniscotibial ligament with a new #11 scalpel blade. For specimens in group II, the medial meniscal release was performed via transection of the medial caudal meniscotibial ligament with a new #64 Beaver blade. Instruments were removed and the specimen was transferred for dissection.

### Arthroscopy

Stifle joints were distended with 0.9% saline solution using a 22 g needle introduced into the lateral parapatellar space. A three-portal arthroscopy method with medial egress canula was performed as previously described by Whitney (21). Fluid ingress via an intravenous pressure bag was used to provide continuous joint distension. Stifle joint arthroscopy was performed using a 2.7 mm 30° oblique arthroscope with video capture system (Stryker 988 Medical Video Camera, 3-chip, Stryker, Kalamazoo, Michigan) with an associated cannula (J-Lock Cannula, Stryker) for all arthroscopic procedures. Infrapatellar fat debridement was performed as needed with a 3.5 mm aggressive shaver (Formula Shaver and CORE Arthroscopic Shaver System, Stryker). The CrCL was assessed and transected using a combination of a previously used meniscal hook knife (Arthrex® Vet Systems) and the arthroscopic shaver. Joint exploration was then performed as follows: proximal compartment (patella and trochlear groove), lateral joint pouch, medial joint pouch, intercondylar notch lateral articular compartment and medial articular compartment. Medial meniscal probing was performed with a small meniscal probe (Small Joint Hook Tip Probe, Arthrex® Vet Systems). For specimens in group III, a separate portal was established to introduce the small Hohmann retractor, which was utilized to translate the tibia forward and improve visualization of the caudal horn of the medial meniscus. The medial meniscal release was performed via transection of the medial caudal meniscotibial ligament with the meniscal hook knife. The transected meniscus was inspected using the meniscal probe. For specimens in group IV, the medial meniscal release was performed in the same manner but without the use of the Hohmann retractor. Valgus stress forces were applied to the limb by an assistant for joint distraction, if necessary for visualization.

### Postsurgical joint examination

All postsurgical joint dissection and examinations were performed by an ACVS resident (DG). Soft tissue structures were excised from the limb using a #10 scalpel blade, leaving the stifle joint and immediate periarticular structures intact. The stifle joint was carefully dissected by one examiner using a #11 scalpel blade and Metzenbaum scissors to carefully remove joint capsule, patella, and fabellae. The medial and lateral collateral ligaments were transected at their origin on the femur.

### Soft tissue structure assessment

The medial meniscal release efficacy was determined by displacing the caudal pole of the medial meniscus via caudomedial traction, evaluating if transection was complete, and measuring the gap created using calipers during maximal displacement. The medial meniscus and caudal cruciate ligament (CdCL) were macroscopically examined for iatrogenic damage. Iatrogenic damage to the CdCL was subjectively evaluated and assigned a score based on severity (1 = mild, 2 = moderate, 3 = severe). Mild damage to the CdCL was characterized as lesions that were ≤ 1 mm in length and partial thickness through the CdCL. Moderate lesions that were longer than 1 mm in length or multiple lesions were present but were all partial thickness. Severe lesions were characterized as lesions ≥1 mm, multiple lesions present, full thickness lesions, or lesions with frayed edges. The medial meniscus, CdCL, collateral ligaments were then dissected from the tibia. Photographs of all aspects of the stifle joint were acquired during each step of dissection. The articular surfaces of the femur and tibia were wrapped in saline soaked gauze to maintain cartilage integrity, while pictures and measurements were obtained.

### Articular cartilage assessment

To quantify iatrogenic articular cartilage damage, India ink assays were performed (15–17, 22). The articular cartilage for each stifle joint was divided into three regions: (1) femoral trochlea and trochlear groove, (2) femoral condyles, and (3) tibial plateau. Photographs were obtained of the stained articular cartilage surfaces with a ruler for magnification calibration. The collected images were analyzed using image software (BIOQUANT Image Analysis Corporation, Nashville, TN). In BioQuant, the normal articular cartilage was manually traced, and total surface area was obtained in mm^2^. All articular cartilages lesions with uptake of India ink were considered iatrogenic. All lesions were manually traced to obtain total lesion surface area in mm^2^, and then calculated as a percentage of total articular cartilage area. The data was exported into a spreadsheet and normalized by the animal’s body weight. Since cadavers used in this study were not fixed for frozen sections at the time of humane euthanasia, and underwent a freeze–thaw cycle, histology of the lesions was not performed.

### Statistical analysis

All analyses were performed by a biostatistician using SAS 9.4 (Cary, NC). A significance threshold of 0.05 was used. Due to small counts, Fisher’s exact tests were performed to test if success or meniscal damage rates differed between procedures.

Damage to the CdCL on an ordinal scale (none, mild, moderate, severe) was compared between procedures with a generalized linear mixed effects model (GLMM) to take into account any correlation between the two limbs within the same animal. The GLMM with a multinomial distribution and a cumulative logit link function (i.e., analogous to ordinal logistic regression) included a fixed factor for procedure and a random intercept for each dog. Multiple comparisons were adjusted for using the Holm-Sidak method. Satterthwaite degrees of freedom method and residual pseudo-likelihood estimation were used.

Cartilage damage in each of the three locations separately were compared between procedures with a linear mixed effects model (LLM) to account for any correlation between the two limbs within the same animal. Histograms and Q-Q plots were examined to evaluate the assumption of normality. The data were right-skewed. Log-transformation of the cartilage damage data resulted in normally distributed residuals. Each univariable LMM included a fixed factor for procedure and a random intercept for each dog. Multivariable LMM included additional covariates of weight and cartilage area and a fixed factor of surgeon (2 different surgeons; JA, BH). Satterthwaite degrees of freedom method and REML estimation were used.

## Results

### Study subjects

Twenty-four dogs were included in the study, resulting in 48 pelvic limbs and 12 stifle joints per study group (6 left and right joints each). One dog was removed from the study due to pre-existing bilateral femoral condyle osteophytosis found during joint exploration. Of the included dogs, bodyweight ranged between 19 and 37.5 kg with a mean weight of 26 kg; 5 pairs were from female and 19 pairs were from male dogs. Breeds represented include 17 Pit Bull Terriers, and 1 of each of the following: Golden Retriever, Boxer, German Shepherd, German Shorthaired Pointer, Malamute, Labrador Retriever, and a mixed breed dog.

### Efficacy of the release

**Figure 1:**
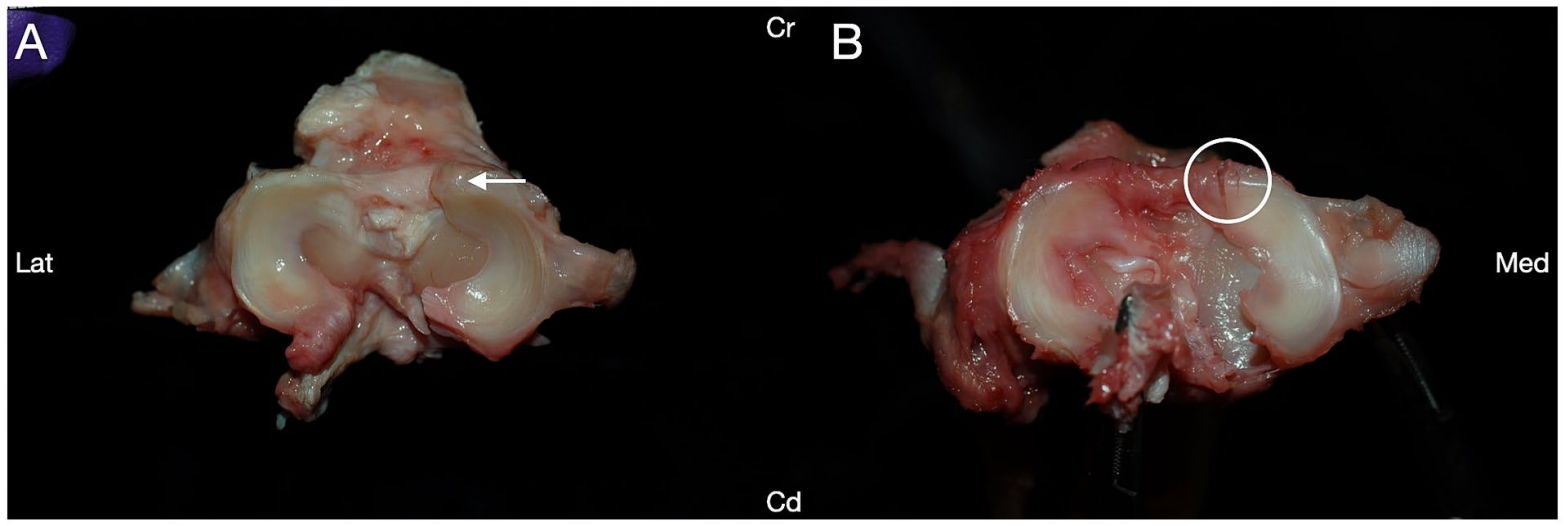
Medial meniscal release procedures were performed via transection of the medial caudal meniscotibial ligament (arrow) in canine cadaveric stifle joints to assess efficacy of multiple release methods. **(A)** Successful transection of the medial caudal meniscotibial ligament was performed in 44/48 stifles. **(B)** The caudal portion of the medial caudal meniscotibial ligament was incompletely transected (arrowhead) in 4/48 dogs. Cranial (Cr); Caudal (Cd); Lateral (Lat); Medial (Med).

Medial meniscal release was successfully completed in 44/48 (91.6%) stifle joints, with no significant difference in efficacy between any groups (*p* = 0.89, Fisher’s exact test) (Figure 1A). In group I, 12/12 (100%) stifles had a complete meniscal release. Incomplete meniscal release was identified in 1/12 (8.3%) stifles in group II, 2/12 (16.7%) stifles in group III, and 1/12 (8.3%) stifles in group IV. In all cases of incomplete transection, the most caudal aspect of the medial caudal meniscotibial ligament remained intact (Figure 1B), and none of the incomplete meniscal releases occurred in paired leg samples from the same dog.

### Iatrogenic medial meniscus damage

**Figure 2:**
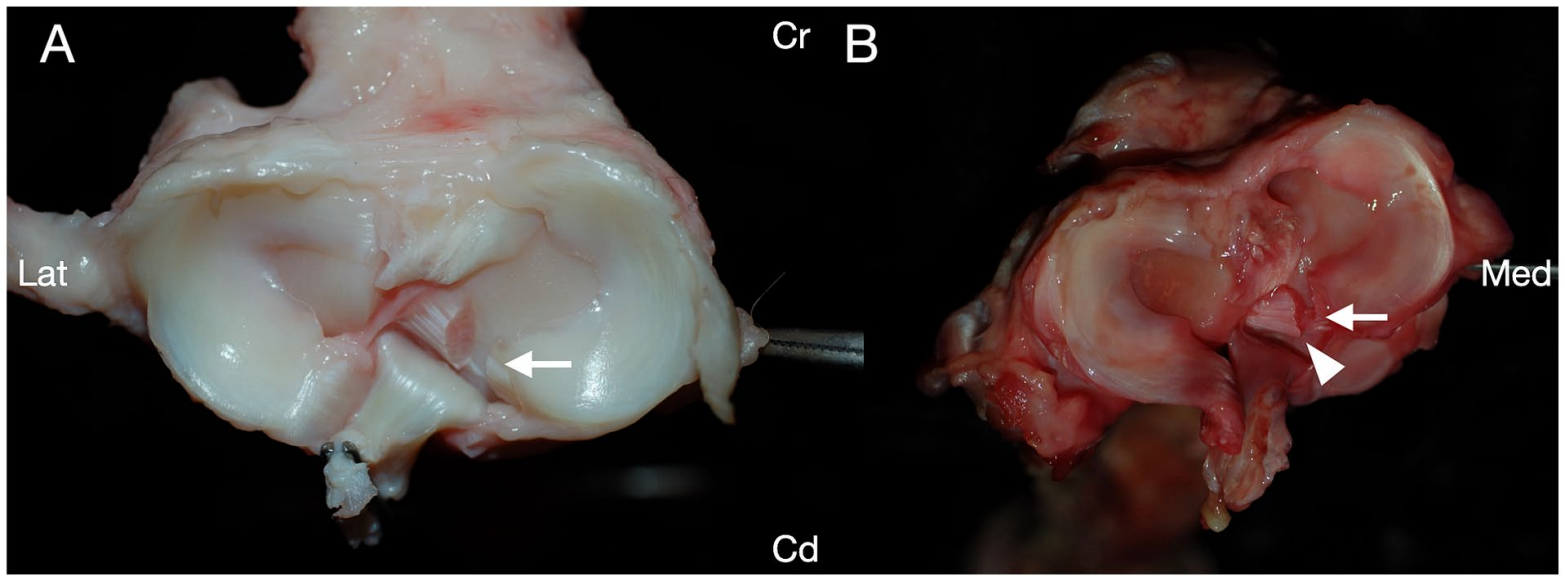
Transection of the medial caudal meniscotibial ligament was performed in canine cadaveric stifle joints, and the structures of the stifles were evaluated for iatrogenic damage caused during the procedure. Iatrogenic damage to the cranial pole of the medial meniscus was noted in 6/48 stifles. **(A)** Each of five stifles had a single laceration to the cranial pole of the medial meniscus (arrow). **(B)** One stifle had multiple lacerations to the cranial pole of the medial meniscus (circle). Cranial (Cr); Caudal (Cd); Lateral (Lat); Medial (Med).

Iatrogenic damage to the cranial pole of the medial meniscus was identified in 6/48 (12.5%) of the stifles, with no difference in iatrogenic meniscal damage between any groups (*p* = 0.48, Fisher’s exact test; Table 1). Iatrogenic meniscal damage was only observed in 1/6 (17%) of the stifles with incomplete meniscal release. Iatrogenic meniscal damage was observed in 3/12 (25%) stifles in group I, 1/12 (8.3%) stifles in group II, and 2/12 (16.7%) stifles in group III. No damage to the cranial pole of the medial meniscus was identified in group IV. Iatrogenic damage to the medial meniscus most commonly presented as a single partial to full thickness laceration across the medial cranial meniscotibial ligament; however, in one stifle within group I, multiple small partial thickness lacerations were identified (Figure 2).

**Table 1:** Presence of iatrogenic medial meniscal damage by stifle visualization group.

Group	Iatrogenic meniscal damage		
	Yes	No	Total
I	3 (25%)	9 (75%)	12
II	1 (8.33%)	11 (91.67%)	12
III	2 (16.67%)	10 (83.33%)	12
IV	0 (0.0%)	12 (100%)	12
Total	6	42	48

Group I, arthrotomy with a Hohmann and meniscal release with a #11 blade; Group II, arthrotomy with a Hohmann and meniscal release with a #64 Beaver blade; Group III, arthroscopy with a Hohmann and meniscal release with a meniscal hook knife; Group IV, arthroscopy with no joint translation and meniscal release with a meniscal hook knife.

### Iatrogenic articular cartilage damage

**Figure 3:**
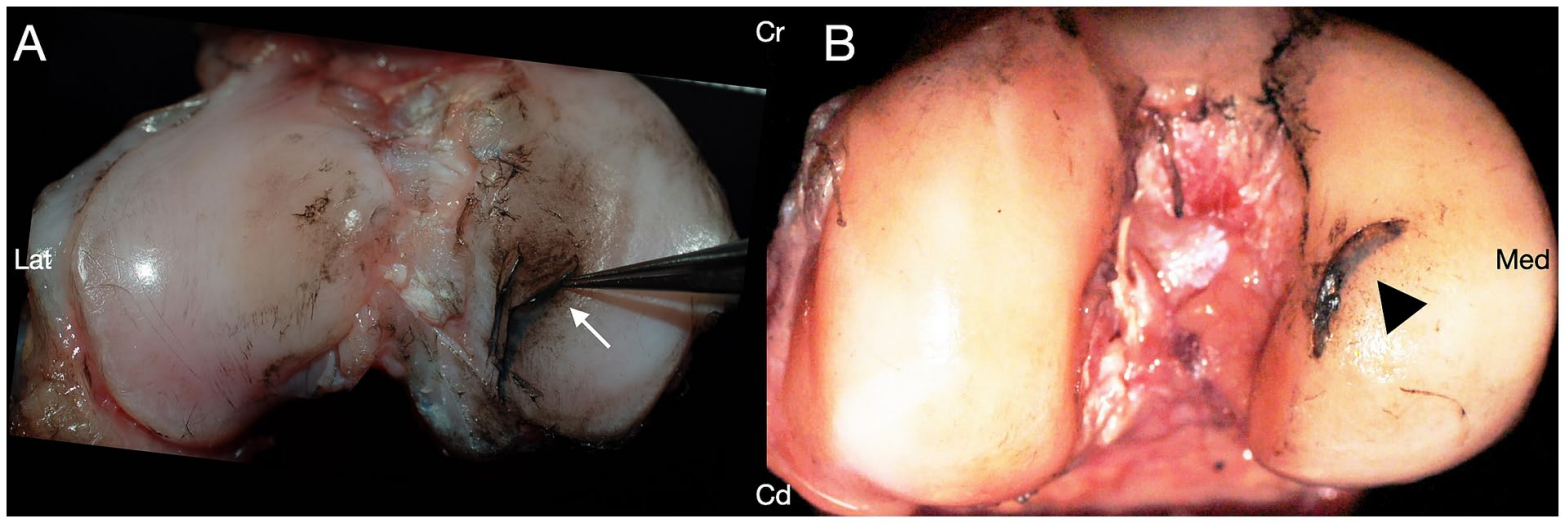
India ink was applied to the cartilaginous articular surfaces of canine cadaveric stifle joints treated with caudal medial meniscotibial ligament transection. Iatrogenic damage was present on at least one surface of the stifle joint in 47/48 stifles. **(A)** India ink uptake revealing iatrogenic cartilage defects of the articular surface adjacent to the medial caudal meniscotibial ligament. **(B)** India ink uptake revealing a deep iatrogenic laceration with cartilage flap on the medial femoral condyle. Cranial (Cr); Caudal (Cd); Lateral (Lat); Medial (Med).

Iatrogenic articular cartilage damage was identified in at least one of the three examined regions in 47/48 (97.9%) of stifle joints (Table 2; Figure 3). One stifle joint in group I had no iatrogenic cartilage damage. GLMM analyses of saturated models suggested in each instance that no main or interaction effects were significant in any model for limb side, sex, or surgeon (all *p* > 0.05), and hence only treatment method was maintained in the final models for comparisons among treatment groups.

**Table 2:** Distribution of iatrogenic articular cartilage damage related to four procedural methods of medial meniscotibial ligament transection in cadaveric dog stifles.

Group	Median (IQR) (%)	Median (IQR): Normalized (mm^2^)	*p*-value	
			Univariable LMM	Multivariable LMM
Femoral Trochlea				
Group 1: Arthrotomy with #11 blade	30 (23–41)	1.17 (0.86–1.76)	0.32	0.34
Group 2: Arthrotomy with #64 Beaver blade	17 (11–30)	0.68 (0.41–1.07)		
Group 3: Arthroscopy with Hohmann retractor	33 (12–64)	1.15 (0.49–2.24)		
Group 4: Arthroscopy (no retractor)	15 (11–45)	0.56 (0.42–1.53)		
Femoral Condyles				
Group 1: Arthrotomy with #11 blade	25 (15–35)	0.94 (0.59–1.43)	0.54	0.49
Group 2: Arthrotomy with #64 Beaver blade	15 (6–24)	0.67 (0.21–1.04)		
Group 3: Arthroscopy with Hohmann retractor	19 (11–30)	0.66 (0.37–1.16)		
Group 4: Arthroscopy (no retractor)	4 (0–15)	0.19 (0–0.55)		
Tibial Plateau				
Group 1: Arthrotomy with #11 blade	13 (3–27)	0.57 (0.14–1.13)	0.28	0.11
Group 2: Arthrotomy with #64 Beaver blade	0 (0–10)	0 (0–0.37)		
Group 3: Arthroscopy with Hohmann retractor	19 (7–31)	0.61 (0.27–1.19)		
Group 4: Arthroscopy (no retractor)	9 (5–16)	0.35 (0.2–0.72)		

No main or interaction effects were significant for limb side, sex, or surgeon (all *p* > 0.05). LMM, linear mixed models; multivariable LMM included additional covariates of weight, cartilage area, and surgeon.

There were no differences noted in total surface area of articular cartilage damage between groups in any of the three regions of the stifle joint (Table 2). The majority of damage to the articular cartilage of the femoral aspect occurred in the area of the medial condyle above the caudal meniscotibial ligament (i.e., near the region of manipulation of the medial caudal meniscotibial ligament), followed by the region of the intercondylar notch. Articular cartilage lesions of the tibial aspect of the stifle joint, seen in 34/48 (70.8%) were localized to the medial tibial condyle, primarily in the region of the medial caudal meniscotibial ligament.

### Iatrogenic caudal cruciate damage

**Figure 4:**
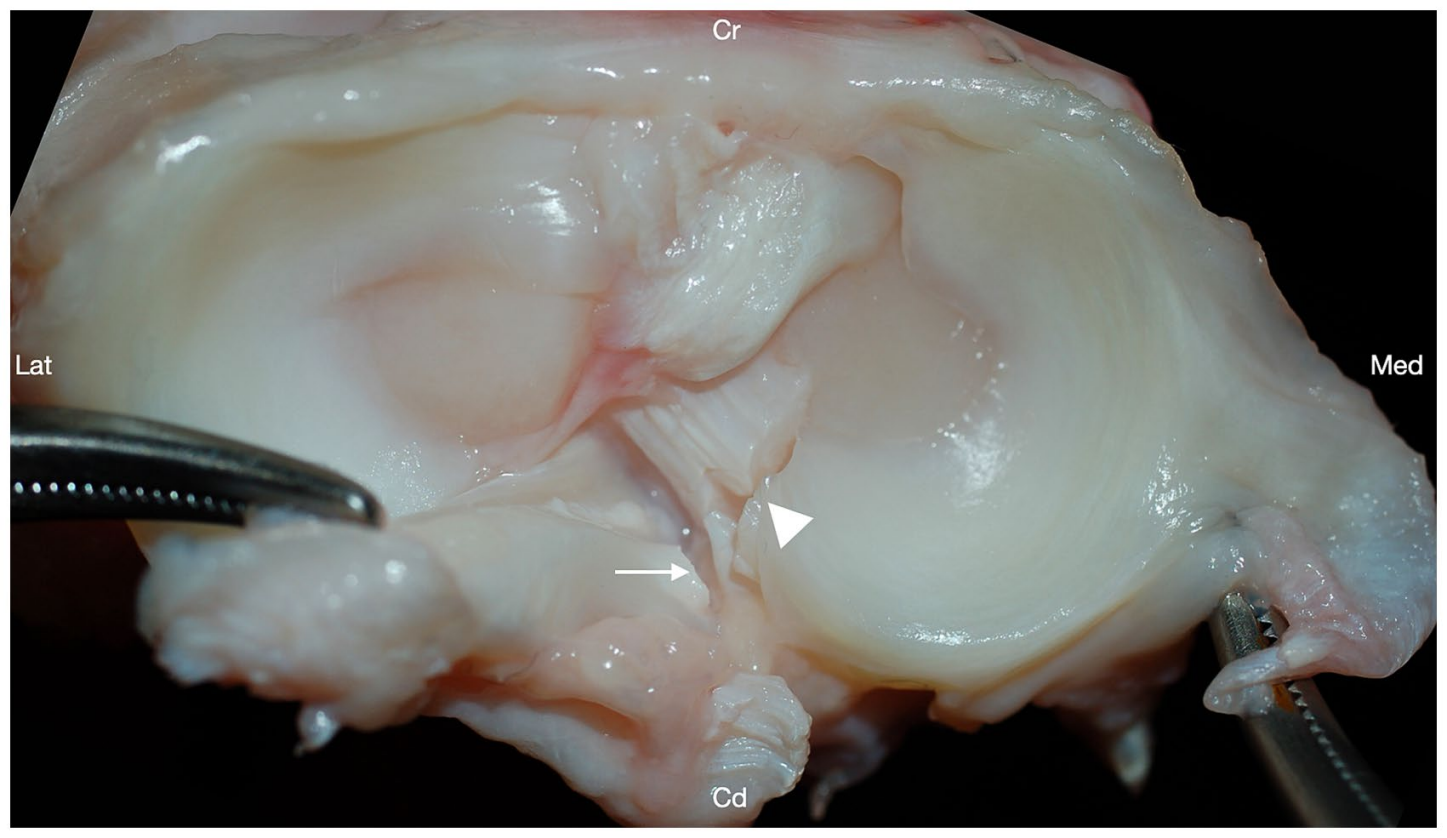
Transection of the medial caudal meniscotibial ligament (arrowhead) resulted in iatrogenic damage to the caudal cruciate ligament (arrow) in 17/48 stifles. Cranial (Cr); Caudal (Cd); Lateral (Lat); Medial (Med).

Iatrogenic damage to the CdCL was identified in 17/48 (35.4%) of the stifle joints, with varying degrees of severity (Figure 4). Iatrogenic damage to the CdCL was identified in 2/12 (16.7%) stifles in group I, 3/12 (25%) stifles in group II, 8/12 (66.7%) stifles in group III, and 4/12 (33.3%) stifles in group IV. Damage to the caudal cruciate was identified at the level of the medial caudal meniscotibial ligament in all damaged cases. Group III stifles had significantly more iatrogenic damage to the CdCL compared to stifles in group I, (*p* = 0.04, GLMM), but there were no other differences between any groups.

## Discussion

Historically the reported increased risk of postliminary meniscal injury following stifle stabilization procedures suggested prophylactic medial meniscal release may be warranted (5–7, 9). However, due to the role of the meniscal cartilages in the biomechanics of the stifle joint and the development of osteoarthritis following medial meniscal release, prophylactic medial meniscal release should not be performed indiscriminately (23–25). Prophylactic medial meniscal release may be considered depending on the surgeon’s preferred method of stifle stabilization, in cases of where the medial meniscus cannot be fully assessed or the damage portion cannot be accessed, or if the potential need for additional surgical intervention in the event of a postliminary medial meniscal tear is not acceptable to the client (2, 26–31). In this study, four methods of performing medial meniscal release were evaluated to identify the most consistent and least harmful approach. Medial meniscal release by transection of the medial caudal meniscotibial ligament was successfully performed in 44 of the 48 stifle joints and no group was statistically advantageous over the others. Based on these findings, we rejected our hypothesis that arthroscopic techniques would result in greater efficacy compared to non-arthroscopic techniques. We also rejected our hypothesis that arthroscopic techniques for medial meniscal release would result in less iatrogenic articular cartilage damage and iatrogenic ligament and meniscal damage compared to non-arthroscopic meniscal release techniques.

Arthroscopy is considered more accurate for diagnosis of medial meniscal pathology than arthrotomy due to the magnification and illumination of the meniscus (12). However, three of the four stifles with incomplete medial meniscal release identified in this study were performed with arthroscopy rather than arthrotomy, which may suggest that improved visualization of the meniscus does not improve accuracy of transection across the entire intended structure. A potential contributing factor to this finding was the use of a previously used meniscal hook knife. While this is clinically appropriate because many practices reuse arthroscopic equipment, this may have contributed to iatrogenic damage to the CdCL and incompleteness of medial meniscal release due to blunting of the knife.

Iatrogenic damage to the remaining medial meniscus was rare in all groups, with no statistical significance between groups. Damage to the cranial pole of the medial meniscus presented a radial incision ranging from complete transection to partial thickness injury and located within the red-red zone (the most peripheral and vascularized zone) and extending from the red-red zone to the white-white zone (the inner and avascular portion of the meniscal cartilage) (32). The meniscal cartilages bear between 40 and 70% of the load across the stifle joint (33,34). In human knees, it is accepted that the meniscal cartilages have poor vascularity beyond 1 to 2 mm from the meniscosynovial junction (33). Therefore, the meniscus lacks the capacity to self-heal, necessitating surgical intervention when injured. Similarly, canine meniscal tissues have an inconsistent blood supply, with vessels originating in the peri-meniscal capsular and synovial tissues and penetrating the peripheral 15 to 25% of the meniscal tissues (32). While complete midportion transection of the medial meniscus was found to heal with fibrovascular scar tissue, longitudinal incisions in the avascular portion of the meniscus failed to heal (32). To the authors knowledge, the effects of damage to the cranial pole of the medial meniscus during medial meniscus release has not been elucidated.

Canine articular cartilage has limited healing capacity as previously demonstrated in the groove model of osteoarthritis (34), therefore developing techniques to decrease iatrogenic articular cartilage damage should be a priority. Rogatko et al. showed that arthroscopic evaluation of the stifle joint resulted in 93% of stifles having iatrogenic articular cartilage damage, but stifles examined with arthrotomy only resulted in articular cartilage damage in 29% of stifles, which suggested that, while arthroscopy may provide better visualization of the stifle joint, it may simultaneously cause more damage than visualization via arthrotomy (16). However, this study found that multiple methods of both arthroscopic and arthrotomy visualization of the stifle joint resulted in iatrogenic articular cartilage damage, in all but one stifle undergoing arthrotomy. This discrepancy in iatrogenic cartilage damage in arthrotomy cases compared to those described by Rogatko et al. may partially be attributed to the use of a small Hohmann retractor in this study and a Venture stifle thrust lever used by Rogatko et al. Regardless of visualization method, care must be taken to prevent damage to the articular surfaces during meniscal assessment and treatment of medial meniscal injuries.

Biomechanical changes associated with the TPLO procedure transform cranial drawer into caudal drawer and increases strain on the CdCL, making it the primary stabilizer of the stifle joint (35–38). Damage to the caudal cruciate resulting from medial meniscal release may be exacerbated by the increased load on the caudal cruciate ligament following stabilization with tibial plateau leveling procedures. While CdCL may be a less important dynamic stabilizer of the intact stifle joint, CdCL rupture in the CrCL-deficient stifle stabilized by tibial plateau leveling procedures may result in recurrent stifle instability. Iatrogenic CdCL damage during medial meniscal release was previously reported in small breed dogs, both with and without the use of a stifle distractor (19). Kim et al. theorized iatrogenic CdCL damage may have occurred due to lack of experience with the technique and failure to meticulously manipulate the meniscal hook knife. Iatrogenic damage to the CdCL occurred in all study groups but was most prevalent and most severe when arthroscopic medial meniscal release was performed with the aid of a small Hohmann retractor. The authors suspect that the position of the Hohmann retractor in the intercondylar notch trapped the CdCL closer to the caudal meniscotibial ligament and in the path of the meniscal hook knife.

In this study, all stifle joints were free of osteoarthritic changes typically associated with CrCL injury. Additionally, all cranial cruciate ligaments were completely transected, and the joints might have been more easily manipulated for medial meniscal release compared to a chronically affected joint. It is possible that the results of this study may not translate to cases where surgeons may choose not to completely transect the intact portion of partially ruptured CrCL during stifle evaluation. A follow-up study evaluating the methods described in groups I-IV for evaluating and surgically treatment meniscal injuries in dogs with partially intact CrCL may be useful.

The cadaveric nature of the study introduced some limitations. The tissues of the stifle, including the medial meniscus, articular cartilage, and caudal cruciate ligament may potentially be more friable after undergoing a freeze/thaw cycle, contributing to the ease of producing iatrogenic damage. Thus, iatrogenic damage may be overrepresented in this study. A study evaluating iatrogenic damage following arthrotomy and arthroscopy using postoperative high-field MRI in clinical cases may be useful for evaluating the impact of these techniques on living tissue. Stifle joints included in this study did not have any evidence of osteophytosis or other signs of the stifle pathology during joint exploration, though this was not confirmed with MRI prior to the study. It is feasible that some damage to the cartilage not overtly visible during inspection already existed. Another weakness is that this study used stifle joints unaffected by osteoarthritic changes occurring with cranial cruciate ligament injuries. After completion of the study, a power calculation was performed using the data collected regarding iatrogenic meniscal damage. The results suggested that 46 stifles per group would be needed to demonstrate the largest difference found between groups (group B: 17% meniscal damage; group C: 0% meniscal damage). Future studies should focus on further exploration of our preliminary findings using a larger sample size. Finally, a meniscal hook knife was used for all arthroscopic meniscal release procedures due to the surgeons’ experience and familiarity with that method. It is possible that the use of a push knife instrument could impact outcomes, and a follow up study evaluating hook knife and push knife outcomes would be useful.

In conclusion, arthroscopy and arthrotomy did not differ when performing medial meniscal release by transection of the medial caudal meniscotibial ligament. Arthroscopic evaluation without joint translation was minimally advantageous in preventing iatrogenic damage to the CdCL. The decision to perform a medial meniscal release will likely remain a controversial one, balancing preservation of the function of the medial meniscus with prevention of future injury to the medial meniscus. Medial meniscal release should be considered when the risk of postliminary tear is high or the prospect of revision surgery to address postliminary medial meniscal injury is not acceptable to the client. If medial meniscal release is performed using a Hohmann retractor, care should be taken to ensure proper placement of the retractor in the intercondylar notch to ensure the CdCL is not entrapped and in the path of the meniscal hook knife or blade.

## Data Availability

The original contributions presented in the study are included in the article/supplementary material, further inquiries can be directed to the corresponding author.
